# Development and Validation of a Cross‐Dimensional Screening Protocol for Remote Phenotyping Applied to Individuals With 3q29 Deletion Syndrome

**DOI:** 10.1111/jir.70116

**Published:** 2026-05-06

**Authors:** R. M. Pollak, E. Sefik, C. Klaiman, M. K. Harner, D. V. Bishop, J. R. Purcell, T. Irving, C. A. Saulnier, S. Pulver, J. F. Cubells, E. F. Walker, M. M. Murphy, J. G. Mulle

**Affiliations:** ^1^ Department of Psychiatry, Robert Wood Johnson Medical School Rutgers University New Brunswick New Jersey USA; ^2^ Center for Advanced Biotechnology and Medicine Rutgers University New Brunswick New Jersey USA; ^3^ Princeton Neuroscience Institute Princeton University Princeton New Jersey USA; ^4^ Marcus Autism Center, Childrens Healthcare of Atlanta & Emory University School of Medicine Atlanta Georgia USA; ^5^ Department of Pediatrics Emory University School of Medicine Atlanta Georgia USA; ^6^ Neurodevelopmental Assessment & Consulting Services Decatur Georgia USA; ^7^ Department of Human Genetics Emory University School of Medicine Atlanta Georgia USA; ^8^ Department of Psychiatry and Behavioral Sciences Emory University School of Medicine Atlanta Georgia USA; ^9^ Department of Psychology Emory University Atlanta Georgia USA; ^10^ General Internal Medicine University of Colorado Anschutz School of Medicine Aurora Colorado USA

**Keywords:** 3q29 deletion, copy number variant, rare genetic disorder, remote phenotyping, schizophrenia

## Abstract

**Background:**

Advances in genomics have resulted in a rapid expansion of the number of known rare genetic disorders (RGDs). However, the low frequency of RGDs presents a challenge for accurately describing the phenotypic spectrum of a given disorder. Remote phenotyping strategies are uniquely poised to address this knowledge gap. Here, we have piloted remote evaluation of cognitive ability and psychosis spectrum symptoms in 3q29 deletion syndrome (3q29del), a hallmark RGD.

**Methods:**

Individuals with 3q29del (*n* = 21, 57% male, mean age = 14.3 ± 8.6 years) were remotely evaluated using the Penn Computerized Neurocognitive Battery (Penn‐CNB), Peabody Picture Vocabulary Test (PPVT) and the Structured Interview for Prodromal Syndromes (SIPS). Scores were compared with prior in‐person IQ testing results and SIPS scores from our previously published study.

**Results:**

Remote cognitive assessment using the Penn‐CNB and the PPVT accurately captured full‐scale IQ (*r* = 0.710, *p* = 0.001) and verbal IQ (*r* = 0.637, *p* = 0.003), respectively, as compared with in‐person assessment with gold‐standard instruments. Psychosis spectrum symptoms measured using the SIPS were significantly correlated between in‐person and remote evaluations (total score *r* = 0.753, *p* = 0.003; positive domain score *r* = 0.806, *p* = 0.0009).

**Conclusions:**

Based on the successful pilot, we designed a protocol for remote phenotyping of individuals with 3q29del. The phenotyping battery is comprised of caregiver‐report and direct assessments to capture the spectrum of neurodevelopmental, neuropsychiatric and medical features associated with the 3q29 deletion. While we focused on specific areas of concern for 3q29del, the high degree of phenotypic overlap between 3q29del and other RGDs renders this protocol amenable for implementation across a variety of RGDs, facilitating a deeper understanding of the phenotypic spectrum and cross‐disorder comparison. Ultimately, we hope that the increased utilization of remote phenotyping strategies will help to expand our understanding of RGDs at large, which will lead to improved clinical management strategies and better long‐term outcomes for affected individuals and their families.

## 1 Introduction

Rapid advancements in genomic technologies have led to the discovery of an increasing number of rare genetic disorders (RGDs) associated with neuropsychiatric and medical outcomes (Kaminsky et al. 2011; Malhotra and Sebat 2012; Coe et al. 2019; Ramoni et al. 2017). RGDs include single‐gene disorders as well as recurrent copy number variants (CNVs), caused by the deletion or duplication of large sections of DNA. It is critical to understand the phenotypic spectrum of a given RGD so that clinicians and their patients are empowered to seek optimal treatment and management strategies. Despite this need, phenotyping efforts for RGDs are fraught with challenges. The low population prevalence of RGDs is a barrier to phenotypic description (Stefansson et al. 2014), as individual clinicians will rarely encounter a critical number of cases in a single clinic (Sullivan and Owen 2020). Aggregate data from clinical case reports may provide valuable information about clinical manifestations (Cox and Butler 2015; Fu et al. 2021; Mudigoudar et al. 2017; Pinchefsky et al. 2017); however, phenotyping in such reports is rarely systematically applied, and ascertainment and publication bias may lead to only the most severe cases being reported (Nissen and Wynn 2014). Large, de‐identified case–control studies of specific disorders such as schizophrenia can provide strong statistical evidence for associations between CNVs and specific diagnoses (Marshall et al. 2017; Rees et al. 2016; Szatkiewicz et al. 2014). However, these studies lack data on the broader phenotypic spectrum, the prevalence of comorbidity or the impact of the variant on individuals without a given psychiatric diagnosis. Specialty clinics or research centres allow for in‐person standardized evaluations conducted by trained clinicians and provide valuable high‐quality phenotypic data (Sanchez Russo et al. 2021; Campbell et al. 2018), but these are only available for select RGDs, come with high operating costs, and impose a significant burden for participants, including costs for flights, hotel and time off from work and school. There is a need to democratize strategies for describing the phenotypic spectra of RGDs.

One such RGD, 3q29 deletion syndrome (3q29del), is caused by a recurrent 1.6 Mb deletion on the long arm of chromosome 3 (hg19, chr3:195 725 000–197 350 000) (Ballif et al. 2008; Glassford et al. 2016; Willatt et al. 2005). The 3q29 deletion is extremely rare, with an estimated population prevalence of 1:30000 individuals (Kendall et al. 2017; Stefansson et al. 2014). Case reports have documented a high prevalence of neuropsychiatric and developmental disorders, including autism spectrum disorder (ASD), anxiety and schizophrenia, as well as medical challenges, including heart defects and failure to thrive (Ballif et al. 2008; Cox and Butler 2015; Glassford et al. 2016; Willatt et al. 2005). Large cohort studies of people with schizophrenia have reinforced the association between 3q29del and schizophrenia, with the 3q29 deletion conferring an estimated > 40‐fold increased risk of schizophrenia (Kirov et al. 2012; Marshall et al. 2017; Mulle 2015; Mulle et al. 2010; Szatkiewicz et al. 2014). To gain a more complete understanding of the phenotypic spectrum of 3q29del, our team previously performed in‐person deep phenotyping in a limited cohort of 32 study participants (Murphy et al. 2018; Sanchez Russo et al. 2021). While the insights from our prior study were impactful, we were only able to describe the most common syndromic manifestations. In addition, despite our best attempts to recruit a representative sample, we cannot rule out that the burden of travel introduced a selection bias in our study.

The COVID‐19 pandemic served to accelerate the development and implementation of telemedicine and remote research strategies (Solomon and Soares 2020; Stroupková et al. 2024; Katakis et al. 2023) that are ideally suited to address the inherent challenges of phenotyping RGDs. Recent studies of multiple RGDs highlight the feasibility of remote phenotyping strategies in largely paediatric cohorts with co‐occurring developmental delay and intellectual disability (Silver et al. 2025; Levy et al. 2022; O'hora et al. 2022). A separate study of the Bio*Me* biobank successfully administered remote clinical and cognitive assessments to adults with schizophrenia or a neurodevelopmental CNV (Zaks et al. 2025). Remote assessment is also gaining popularity in the evaluation of children with idiopathic ASD, with studies showing high concordance between remote and in‐person assessments (Katakis et al. 2023; Stroupková et al. 2024). Together, these data highlight the validity of remote assessment and its utility in accessing large cohorts of patients with a rare disorder. However, it is unknown whether these strategies will be effective in 3q29del. Here, we report a validated, comprehensive remote phenotyping protocol designed to capture the full phenotypic spectrum of 3q29del.

## 2 Methods

### 2.1 Prior Clinical Evaluation

In our prior in‐person phenotyping study, 34 individuals with 3q29del travelled to Atlanta, Georgia, for assessments at the Marcus Autism Center (affiliated with Emory University and the Children's Hospital of Atlanta [CHOA]). Study participants were evaluated over 2 days using a transdiagnostic deep‐phenotyping battery with gold‐standard instruments administered by an expert clinical team, as described previously (Murphy et al. 2018; Sanchez Russo et al. 2021). As a part of this protocol, cognitive ability was assessed using the Differential Ability Scales, 2nd ed. (DAS‐II) (Elliott et al. 1990) in subjects under 18 years of age (*n* = 26) or the Wechsler Abbreviated Scale of Intelligence, 2nd ed. (WASI‐II) (Wechsler 1999) in subjects 18 years of age and older (*n* = 8). Prodromal symptoms of psychosis were assessed using the Structured Interview for Psychosis‐Risk Syndromes (SIPS) in all participants 8 years of age and older (*n* = 24) (Miller et al. 2003).

### 2.2 Participants

To evaluate the feasibility of remote phenotyping in individuals with 3q29del, we conducted an abbreviated remote battery with individuals who had also participated in our in‐person phenotyping study (Murphy et al. 2018, Sanchez Russo et al. 2021). Of 34 eligible participants, 21 individuals (57% male, mean age = 14.3 ± 8.6 years; 61.8% response rate) (Table 1) completed the abbreviated battery (mean time between assessments = 2.0 ± 0.9 years). The subset of original participants that completed the remote battery was representative of the original study population; there were no significant differences between the groups for sex, age or IQ (all *p* > 0.05, Table S1).

**TABLE 1:** Description of study sample that participated in remote phenotyping pilot.

		Mean ± SD	Range
Age (years)		14.33 ± 8.53	6–40
Composite IQ		74.48 ± 11.05	43–96

		*N*	%
Sex			
	Male	12	57.14%
	Female	9	42.86%
Diagnosis			
	ASD	5	23.81%
	Intellectual disability	6	28.57%
	Graphomotor weakness	15	71.43%
	Anxiety	10	47.62%
	Prodrome/psychosis	3	14.29%
	ADHD	13	61.90%

### 2.3 Pilot Design

The pilot remote battery focused on receptive vocabulary, assessed using the Peabody Picture Vocabulary Test (PPVT‐5) (Dunn 2019); cognitive ability, assessed using the Penn Computerized Neurobehavioral Test Battery (Penn‐CNB) (Moore et al. 2015); and psychotic symptoms, assessed using the Structured Interview for SIPS (Miller et al. 2003). Evaluators were trained on the administration of all assessments and certified for use prior to enrolling participants. Evaluators were blinded to prior in‐person testing results when completing evaluations.

After remote informed consent, each participant met via a HIPAA‐compliant videoconferencing platform (Zoom) with a trained research assistant for three remote study visits, lasting between 30 and 90 min each. The researcher administered the PPVT‐5, Penn‐CNB and SIPS at the first, second and third remote study visits, respectively. A standard protocol was in place to assess suicide and self‐harm risk, with clinical supports in place for further assessment if needed.

### 2.4 Data Analysis

We treated our prior in‐person assessments as gold‐standard values for comparison. To compare performance between in‐person and remote phenotyping in our cohort, we used Pearson correlation tests performed in R version 4.2.2 (R Core Team 2008). Pearson correlations were chosen because the time gap between the in‐person and remote evaluations introduces expected developmental changes in performance; the goal of the analysis was to test method consistency rather than absolute agreement in scores across evaluations. Remote PPVT‐5 total standardized score was compared with in‐person measures of verbal IQ, remote Penn‐CNB global accuracy score was compared with in‐person measures of full‐scale IQ, and remote SIPS scores were compared with in‐person SIPS evaluation. To test the consistency of the SIPS item‐level performance across evaluations, we performed three additional analyses. First, we implemented a repeated‐measures MANOVA using the MANOVA.RM R package, with permuted *p*‐values to account for the small sample size (Friedrich et al. 2018), to compare the overall SIPS profile shape between timepoints. Second, we calculated a Pearson correlation for each subject's scores between the in‐person and remote evaluations. Finally, we calculated a Pearson correlation for each SIPS item across all study participants, comparing the in‐person and remote evaluations. Data visualization was performed using the ggplot2 R package (Wickham 2009).

## 3 Results

### 3.1 Remote Phenotyping Protocol Validation

**FIGURE 1:**
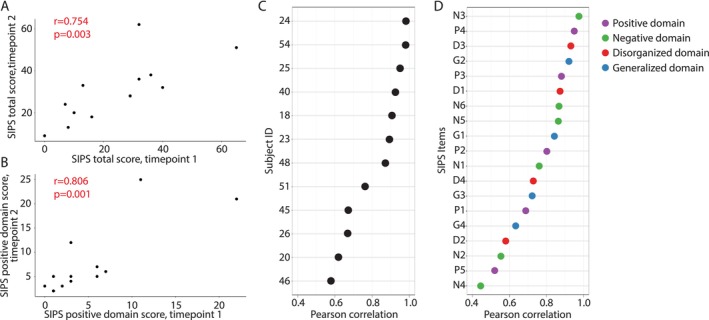
**(A)** Correlation between in‐person (Timepoint 1) and remotely (Timepoint 2) administered SIPS total score. **(B)** Correlation between in‐person (Timepoint 1) and remotely (Timepoint 2) administered SIPS positive domain score. **(C)** Correlation in SIPS scores between in‐person and remotely administered SIPS for each participant. Subject 34 is not shown because their score at the in‐person assessment was 0 (Figure S1), so a correlation could not be calculated. **(D)** Correlation in scores between in‐person and remotely administered SIPS for each SIPS item.

**FIGURE 2:**
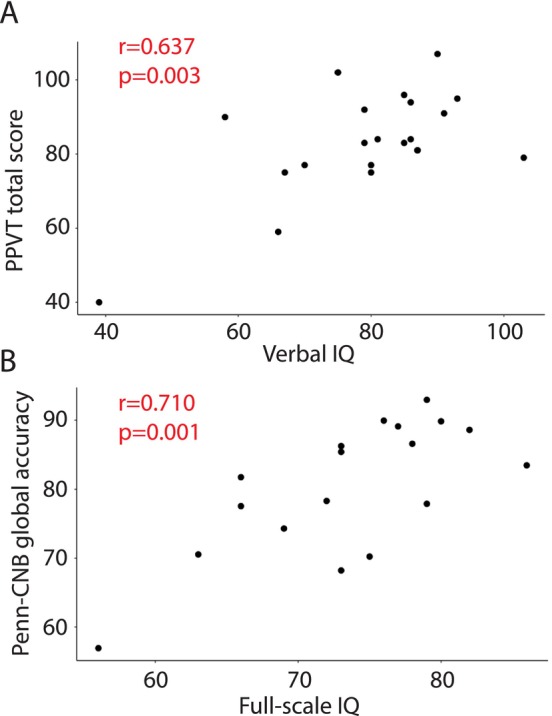
**(A)** Correlation between in‐person gold‐standard verbal IQ and remotely administered PPVT total score. **(B)** Correlation between in‐person gold‐standard full‐scale IQ and remotely administered Penn‐CNB global accuracy score.

To determine whether remote assessments accurately captured phenotypes in individuals with 3q29del, we compared the results of our remote evaluations to prior gold‐standard in‐person evaluations. We found that there was a significant positive correlation between each pair of measures, indicating that the remote assessments are effectively capturing phenotypic information in 3q29del study participants as compared with in‐person assessment (Table 2). The tightest correlation was between the in‐person and remote SIPS measures (Figure 1, Figure S1); the SIPS total score had a correlation of 0.753 between the two measures (*p* = 0.003; Figure 1A), and the SIPS positive domain score had a correlation of 0.806 (*p* = 0.0009; Figure 1B). The SIPS profile shape was not different between the in‐person and remote evaluations (*p* = 0.107), indicating that SIPS performance was consistent between the evaluation modalities. Within each individual, the correlation in SIPS scores between the two measures ranged from 0.574 to 0.977 (Figure 1C), and the correlation between scores for each SIPS item ranged from 0.438 to 0.969 (Figure 1D). Within the cognitive domain, the remote PPVT‐5 and in‐person verbal IQ had a correlation of 0.637 (p = 0.003; Figure 2A), and the remote Penn‐CNB global accuracy score and in‐person full‐scale IQ had a correlation of 0.710 (*p* = 0.001; Figure 2B). Together, these data demonstrate that remote phenotyping in 3q29del yields results consistent with gold‐standard measures.

**TABLE 2:** Comparison of in‐person and remote methods used in pilot remote phenotyping.

Dimension tested	In‐person instrument	Remote instrument	Correlation	*p*
Verbal ability	DAS/WASI	PPVT	0.637	0.003
Cognitive ability	DAS/WASI	Penn‐CNB	0.71	0.001
Psychosis‐risk symptoms	SIPS	SIPS	0.753	0.003

### 3.2 Development of a Comprehensive Remote Phenotyping Protocol for 3q29del

Based on the successful pilot of remote phenotyping in our study population of individuals with 3q29del, we moved to create a comprehensive remote phenotyping battery designed to measure the primary domains and subdomains that are impacted in individuals with 3q29del.

#### 3.2.1 Participants

**FIGURE 3:**
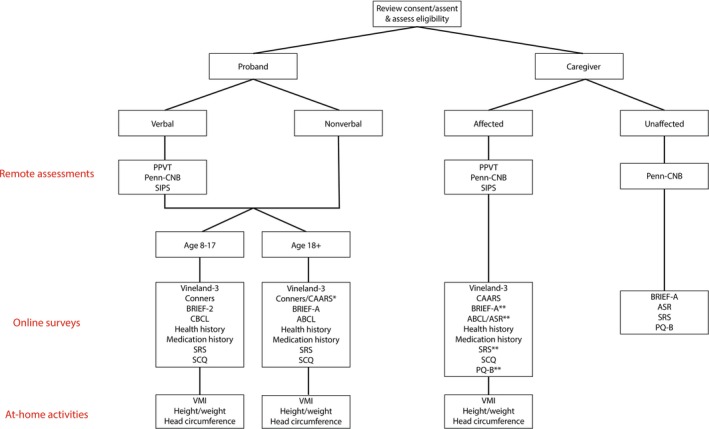
**Flow chart of study design.** *The Conners is administered to adult subjects ages 18–21, while the CAARS is administered to subjects over the age of 21. **Affected caregivers (those with the 3q29 deletion) complete the BRIEF‐A, ASR, SRS, and PQ‐B as self‐report instruments, in addition to the standard subject battery of informant‐report surveys, which are completed by a parent, spouse or other informant.

We will recruit individuals with the 3q29 deletion from the existing online 3q29 Registry (3q29deletion.com) (Glassford et al. 2016), as well as direct referrals and contacts. To be eligible for the study, probands must be 8 years of age or older, fluent in English and have a clinically confirmed diagnosis of 3q29del. Non‐verbal participants will be administered an abbreviated study protocol. Participants and their caregivers must be willing and able to provide informed consent and be fluent in English to participate. Exclusion criteria are non‐fluency in English and age younger than 8 years. As a comparison sample, we will enrol typically developing controls recruited from community sources and online platforms for individuals interested in participating in research (ResearchMatch). All enrollment, consenting and data collection procedures are identical between 3q29del probands and typically developing controls. Familial background has been shown to be a significant modifier of outcomes (Taylor et al. 2023). The flexibility of remote data collection allows us to assess first‐degree relatives (usually parents) to estimate the impact of this in the 3q29 deletion population. The abbreviated battery of instruments completed by caregivers is shown in Figure 3.

#### 3.2.2 Phenotyping Battery

##### 3.2.2.1 Executive Function, Attention and Hyperactivity

Executive function is assessed using the Behaviour Rating Inventory of Executive Function, 2nd Edition (BRIEF‐2) for participants 18 years of age or younger or the adult version (BRIEF‐A) for participants over 18 years of age (Gioia et al. 2015; Roth and Gioia 2005). Caregivers rate behaviours associated with self‐control and problem‐solving skills along nine dimensions of executive functioning: inhibiting distractions, self‐monitoring, shifting, emotional control, initiation, working memory, planning, organization and task monitoring. Subjects aged 21 or younger are also assessed using caregiver‐report on the Conners (Conners‐3 or Conners‐4), a questionnaire designed to assess for DSM‐V symptoms of ADHD, conduct disorder and oppositional defiant disorder (Conners 2022; Conners et al. 2018). This age cutoff was established due to the phenotype of developmental delay frequently observed in those with 3q29del (Glassford et al. 2016; Sanchez Russo et al. 2021; Cox and Butler 2015). Subjects over the age of 21 are assessed for attentional deficits via the analogous Conners' Adult ADHD Rating Scale (CAARS or CAARS‐2) (Conners et al. 1999).

##### 3.2.2.2 Cognitive Ability and Receptive Vocabulary

Receptive verbal comprehension is assessed in subjects at the first study visit using the PPVT‐5, a computerized, norm‐referenced measure of receptive vocabulary based on words in Standard American English (Dunn 2019). On the PPVT‐5, subjects hear a word and select one of four illustrations that best depict the word. The Penn‐CNB is conducted with each subject as a measure of cognitive ability across five domains: executive, episodic memory, complex cognition, social cognition and sensory/motor speed (Moore et al. 2015). The battery consists of 13 computerized tasks and puzzles designed to measure the accuracy and speed of the participants' responses (full list of tasks in Supporting Information). The battery is administered remotely by a certified researcher who provides standardized instruction, ensures participant understanding and takes detailed notes on any issues that may compromise data quality, such as software glitches, interruptions or idiosyncratic participant behaviour (detailed information on score calculation and quality control in Supporting Information, Figure S5).

##### 3.2.2.3 Adaptive Behaviour

Adaptive behaviour is assessed using the Vineland Adaptive Behaviour Scales‐3 Parent/Caregiver form (Vineland‐3), a standardized caregiver‐report assessment of an individual's ability to independently perform age‐appropriate day‐to‐day activities across four domains: communication, daily living skills, socialization and motor skills (Sparrow et al. 2016).

##### 3.2.2.4 Social Function Related to ASD

Social function relevant to ASD is assessed using the Social Responsiveness Scale (SRS), which measures social information exchange across five subscales: social awareness, social cognition, social communication, social motivation and restricted interests and repetitive behaviours (Constantino and Todd 2012). Caregivers complete an informant‐report form on their child's behaviours. Additionally, caregivers complete the Social Communication Questionnaire (SCQ) as a secondary measure of social function in the subject (Rutter, Bailey, and Lord 2003). The SCQ is derived from the Autism Diagnostic Interview‐Revised (ADI‐R) (Rutter, Le Couteur, and Lord 2003), evaluating social behaviour across three domains: reciprocal social interaction, communication and language, and restricted, repetitive and stereotyped interests and patterns of behaviour.

##### 3.2.2.5 Psychosis Risk Symptoms and Psychosis

The SIPS assesses the presence, duration and severity of subthreshold symptoms of psychosis (Miller et al. 2003). The SIPS is a gold‐standard semi‐structured interview used to assess prodromal symptoms of psychosis. Pre‐interview questions are asked at the beginning of the caregivers' remote study visit. The SIPS interview is then conducted by certified interviewers remotely with the subject without the caregiver present, unless there is a strong preference for caregiver attendance for the subject's comfort or intellectual disability. The SIPS is recommended to be conducted with interviewees between the ages of 10 and 45, with an IQ above 70; however, prior work by our team and others has demonstrated that the SIPS can be administered to children as young as 8 years of age provided that the interview is slightly adapted for the developmental level of the subject (Sefik et al. 2024; Tang et al. 2014). The SIPS will be adapted for administration in participants under 10 years of age via the following: simplifying questions, using concrete examples and involving the caregiver to clarify context and ensure understanding. If participants are unable to complete the SIPS with these adaptations, their data will be partitioned out of the final analysis. When necessary, corroborative information is ascertained from a caregiver in a follow‐up interview to resolve questionable information reported by subjects who exhibited difficulty with understanding or answering interview questions. Participants are rated on a scale of 0‐6 across positive, negative, disorganised and general symptom domains, with scores of 3 or greater on any item indicating clinically significant prodromal symptoms.

##### 3.2.2.6 General Psychopathology

General psychopathology is assessed using the Adult Behaviour Checklist (ABCL) for participants 18 years of age or older or the Child Behaviour Checklist (CBCL) for participants younger than 18 years of age (Achenbach and Rescorla 2001; Achenbach and Rescorla 2003). These are standardized, caregiver‐report questionnaires that measure the presence of behavioural and emotional problems across eight syndrome scales: anxious/depressed, withdrawn/depressed, somatic complaints, social problems, thought problems, attention problems, rule‐breaking behaviour and aggressive behaviour.

##### 3.2.2.7 Graphomotor Function

The Beery‐Buktenica Developmental Test of Visual‐Motor Integration—6th edition (Beery VMI) measures visual‐motor integration (Beery and Beery 2010), which is impaired in individuals with 3q29del (mean score = 69.3 ± 16.7) (Pollak et al. 2023). The Beery VMI requires subjects to copy each of 28 abstract designs of increasing complexity. Caregivers are instructed on proper administration of the Beery VMI during their remote study visit, and step‐by‐step written instructions are included in the study kit. Subjects complete the Beery VMI with caregiver oversight outside of study visits (Figure S4). The worksheet is returned with the study kits and scored by the study team.

##### 3.2.2.8 Medical/Physical

A detailed medical history questionnaire (see Supporting Information) is completed online by the caregiver. The survey includes questions to assess for pregnancy history, including diagnostic testing/screening, prescriptions or supplements taken and drug or alcohol exposure; birth history, including complications, birthweight and gestational age; developmental history, including motor and communication milestones; and a systems‐based medical history. The survey has been adapted from our previous study (Murphy et al. 2018). Height, weight and head circumference measurements are collected by caregivers using standardized equipment and procedures (see Supporting Information, Figure S3) to evaluate the physical stature of subjects.

##### 3.2.2.9 Medication History and Efficacy

A lifetime psychiatric medication history questionnaire is also completed by caregivers. A reference list of psychiatric medications is provided (Table S2). For each medication that the subject has taken, either currently or in the past, the caregiver records dosage, duration, indication, side effects and perceived efficacy.

#### 3.2.3 Data Collection Procedures

Data are collected via remote study visits, online self‐report or informant‐report surveys and at‐home data collection by the caregivers with instruction from trained researchers (Table 3). For adult subjects, a spouse or sibling may participate as an informant in lieu of a caregiver. Figure 3 summarizes the study protocol. Once informed consent is obtained, demographic data are collected via a custom REDCap (Harris et al. 2019; Harris et al. 2009) survey, and remote study visits are scheduled using a HIPAA‐compliant online scheduling platform. A study kit including necessary study materials and instructions is mailed to the family (see Supporting Information), and links to online surveys are emailed to the caregivers. Online assessments are administered through REDCap and instrument publishers' websites. Assessments and questionnaires have been selected to measure the primary domains and subdomains that are impacted in individuals with the 3q29 deletion (Sanchez Russo et al. 2021; Glassford et al. 2016). The study kit also includes materials to collect height, weight and head circumference data on the subject (Supporting Information).

**TABLE 3:** **Assessments and domains measured.** Complete list of assessments and surveys to be completed by subjects and caregivers, categorized by domain. Online surveys are completed by caregivers. “Remote administration” assessments are administered over Zoom by a trained researcher. “Administered by caregiver” assessments are administered by a caregiver after receiving instruction from a trained researcher.

Domain/phenotype	Instruments for subjects	Instruments for caregivers	Administration method
**Executive function**	BRIEF‐2/BRIEF‐A (Gioia et al. 2015, Roth and Gioia 2005)	BRIEF‐A (Roth and Gioia 2005)	Online survey
**ADHD**	Conners/CAARS (Conners 2022, Conners et al. 1999, Conners et al. 2018)	—	Online survey
**Cognitive ability**	Penn‐CNB (Moore et al. 2015)	Penn‐CNB (Moore et al. 2015)	Remotely administered
**Receptive vocabulary**	PPVT (Dunn 2019)	—	Remotely administered
**Adaptive behaviour**	Vineland‐3 (Sparrow et al. 2016)	—	Online survey
**Social function**	SRS (Constantino and Todd 2012)	SRS (Constantino and Todd 2012)	Online survey
	SCQ (Rutter, Bailey, and Lord 2003)	—	Online survey
**Psychosis**	SIPS (Miller et al. )	—	Remotely administered
	—	PQ‐B (Loewy et al. 2011)	Online survey
**General psychopathology**	CBCL/ABCL (Achenbach and Rescorla 2001, Achenbach and Rescorla 2003)	ASR (Achenbach and Rescorla 2003)	Online survey
**Visual‐motor integration**	Beery VMI (Beery and Beery 2010)	—	Administered by caregiver
**Medical/physical**	Medical history	—	Online survey
	Height/weight/head circumference	—	Administered by caregiver
**Medications**	Psychiatric medication history	—	Online survey

Subjects meet via Zoom with a trained research assistant for three remote study visits, lasting between 30 and 90 min each. At the first remote study visit, the researcher administers the PPVT‐5 (Dunn 2019) to the proband. The researcher also provides instruction to the caregiver for measuring the subject's head circumference using the provided materials. Head circumference is measured on‐camera with guidance from the researcher to ensure an accurate measurement. At the second and third remote study visits, a researcher administers the Penn‐CNB (Moore et al. 2015) and the SIPS (Miller et al. 2003) to the subject, respectively. A standard protocol is in place to assess suicide and self‐harm risk, with clinical supports in place for further assessment if needed. Non‐verbal study participants do not complete study visits; data collection for these participants is limited to online surveys and at‐home activities (Figure 3). One caregiver is also queried on their child's developmental, social and psychological history, as well as any family history of mental illness.

Upon completion of all data collection instruments, participants return the study kit using an included prepaid shipping label. The research team collates all questionnaire and assessment data into a written research evaluation report to be returned to each family. A follow‐up videoconferencing session is then arranged between the subject/caregivers and the research team, at which point the research evaluation report is emailed to the family, and results are discussed with the subject and/or their caregivers.

## 4 Discussion

Here, we have demonstrated the validity and feasibility of remote phenotyping in individuals with 3q29 deletion syndrome. We further present a protocol for remote evaluation of multidimensional phenotypes associated with 3q29del. By harnessing the advantages of remote evaluation, this protocol allows for the administration of standardized assessments across core domains previously found to be impacted in individuals with 3q29del. The proposed protocol allows for the collection of multidimensional behavioural data at scale, while minimising participant burden and maximising feasibility.

Remote study designs have several important advantages over traditional in‐person clinics when considering RGDs. By definition, remote assessment can be performed anywhere, eliminating the need to travel to a specialty clinic for evaluations. Travel is a significant barrier to care for individuals with RGDs, as it is costly, requires significant absences from school or work, and can be stressful for the affected individual and their family members (Grosse et al. 2009). Additionally, when an individual goes to a specialty clinic to receive care, there are typically numerous sessions and evaluations in a day, which can lead to testing fatigue. Remote studies allow the evaluations to be divided across multiple sessions, reducing the strain on the participant and ensuring that the testing can be completed at times that are convenient for the family's existing schedule and obligations. Finally, remote study designs can serve to expand the spectrum of recruitment for a given RGD—it is possible that the most severely affected individuals are unable to travel, and higher‐functioning individuals may choose not to participate in in‐person studies because they do not require a high level of specialty care. Convenient, at‐home remote assessments can serve the needs of the full spectrum of abilities associated with RGDs and reduce ascertainment bias.

While we specifically designed the protocol presented here for phenotyping 3q29del, it should be noted that it is also generalizable to other RGDs given the high degree of phenotypic overlap, especially among CNV disorders. Developing a consistent, standardized phenotyping battery will facilitate cross‐disorder analysis (Mulle et al. 2021); without core phenotyping instruments, it is difficult or impossible to identify areas of phenotypic convergence and divergence. Indeed, some of the instruments included in the present battery have already been implemented in other disorders; namely, the Penn‐CNB in 22q11.2 deletion syndrome (White et al. 2024) and the SIPS in the North American Prodrome Longitudinal Study of individuals at high risk for psychosis (Addington et al. 2015). Harmonising instruments across studies allows for the well‐powered cross‐disorder analyses that will ultimately lay the groundwork for studies of the mechanisms underlying phenotype development in these RGDs.

Given the high risk for psychosis associated with the 3q29 deletion (Mulle 2015; Kirov et al. 2012; Stefansson et al. 2014), it was critical to validate remote use of the SIPS in our study population. The coexisting cognitive and attentional challenges, as well as high rates of anxiety in individuals with 3q29del (Glassford et al. 2016; Klaiman et al. 2022; Murphy et al. 2020; Sanchez Russo et al. 2021; Cox and Butler 2015; Willatt et al. 2005; Ballif et al. 2008), introduced potential sources of variation that may not be present in populations without a genetic diagnosis. By design, the SIPS probes highly sensitive and personal experiences, and a strong rapport between the participant and interviewer is beneficial when eliciting this information. We were concerned that the study subjects may not feel a connection to the interviewer when meeting remotely and may therefore be inclined to mask or minimize symptoms. The participant's subjective experience of the interview may also be affected by the remote format; meeting an interviewer over Zoom is potentially a very different experience than meeting with the interviewer face‐to‐face in a private room. However, the high correlations and item‐level stability across assessment modalities (Figures 1, S1) indicate that remote administration of the SIPS is valid and elicits high‐quality data in individuals with 3q29del.

The protocol described here is an important step toward a more comprehensive understanding of 3q29del, and ideally a harmonized phenotyping approach for RGDs at large. However, there are important limitations to consider when discussing remote phenotyping. First, the researchers conducting remote phenotyping assessments have significantly less control over the testing environment and possible distractions as compared with an in‐person study. It is also harder for the researcher to make granular behavioural observations, such as observations about eye contact and social cuing. To address these concerns, our researchers take detailed notes during all sessions to document any perceived distractions or interruptions that may compromise data quality. Although remote studies can increase ascertainment across the phenotypic spectrum, individuals with poor internet connection or limited access to technology are unable to participate in remote studies. In our pilot study, we were concerned about biased ascertainment due to technology access. To protect our findings against this possibility, we obtained a study laptop that could be shipped to families that needed it. To our surprise, all study participants had access to a laptop or desktop computer, regardless of socioeconomic status. This is likely a consequence of the pandemic and the rise of remote classroom strategies. Participant retention across multiple study visits is also a concern; however, we have found that families are highly motivated to complete all required assessments in order to receive their final report with the testing results. Indeed, the biggest challenge to participant enrollment and retention that we encountered was scheduling difficulties and finding time for participants to complete assessments; given these challenges, we were still able to retain 61.8% (21 of 34) of participants from our original in‐person phenotyping study. In our pilot study, there was an average of 2 years between assessments, which can introduce variance due to natural developmental changes or symptom progression. However, the high correlations between assessments suggest that we are measuring stable traits that are robust to the modality, the assessment or the tasks used. The time interval in our study is comparable to other studies that used both longitudinal Penn‐CNB and SIPS measurements in Williams syndrome and 22q11.2 deletion syndrome, two phenotypically similar genomic disorders (Gur et al. 2021; Weinberger et al. 2018). Further, it can be difficult to accurately measure cognition remotely for the youngest and most severely affected individuals; the Penn‐CNB is validated for remote use (White et al. 2024) but does require basic verbal comprehension, focus and motor skills to complete the tasks. Our researchers take detailed notes during the Penn‐CNB administration to assess participant understanding of the tasks, idiosyncratic behaviour and reaction time, to ensure that participants understand and are able to complete each task (details in Supporting Information). To supplement the Penn‐CNB, we are also collecting data on verbal ability using the PPVT‐5 (Dunn 2019), and we request any prior cognitive testing reports that participants have. Additionally, our validation analysis shows that both the Penn‐CNB and the PPVT‐5 performance are strongly positively correlated with gold‐standard in‐person IQ measures, which is consistent with prior work in 22q11.2 deletion syndrome demonstrating that the Penn‐CNB global accuracy score is a suitable proxy for IQ testing (Gur et al. 2021). We have observed that, when 3q29 deletion individuals are non‐verbal or have lower cognitive ability, such that they cannot effectively complete the Penn‐CNB, they almost always have alternate cognitive testing results; however, the vast majority of individuals with 3q29del that we have evaluated only present with mild to moderate intellectual disability (Klaiman et al. 2022) and were able to successfully complete the assessment. Finally, some tasks on the Penn‐CNB are sensitive to the state of the participant, especially tasks like the Go/No Go Task and the Continuous Performance Task, whereas traditional IQ testing measures a stable trait. However, the Penn‐CNB as a whole is thought to measure a global trait (Moore et al. 2015; Gur et al. 2021), and the high correlation between full‐scale IQ and the Penn‐CNB global accuracy score (Figure 2B) suggests that we are measuring a stable trait in our study population. Further, excluding the Continuous Performance Task from our Penn‐CNB global accuracy score calculation did not meaningfully change the correlation with FSIQ (from 0.710 in Figure 2B to 0.740 in Figure S2), suggesting that the inclusion of more state‐sensitive tasks did not have a strong influence on our estimation of cognitive ability using the Penn‐CNB.

This is the first report of remote cognitive and behavioural phenotyping in individuals with 3q29del, as well as a description of a standardized remote testing battery for phenotyping 3q29del and phenotypically similar RGDs. It is critical to develop a comprehensive understanding of the phenotypic spectrum of RGDs in order to improve treatment plans and outcomes for affected individuals. Further, expanding our understanding of RGDs such as the 3q29 deletion may also lead to mechanistic insights about the development and pathogenesis of common psychiatric disorders such as schizophrenia and ASD. We hope to inspire other groups to adopt a standardized phenotyping protocol to facilitate deep understanding of individual RGDs as well as comparison across RGDs, which will ultimately benefit clinicians, families and affected individuals by helping to guide care plans and improve treatment outcomes.

## Ethics Statement

This study was approved by Emory University's Institutional Review Board (IRB00064133) and Rutgers University's Institutional Review Board (PRO2021001360).

## Conflicts of Interest

CAS reports receiving royalties from Pearson Assessments for the Vineland‐3. All other authors report no competing interests.

## Funding

This work was supported by National Institute of Mental Health R01 MH110701 (PI Mulle) and R01 MH126449 (PI Mulle).

## Consent

All study subjects gave informed consent prior to participating in this study.

## Supporting information




**Table S1:** Comparison of subjects who participated in remote pilot to subjects who did not participate.
**Figure S1:** SIPS profiles at Timepoint 1 (in‐person evaluation) and Timepoint 2 (remote assessment) for all pilot study participants.
**Figure S2: (A)** Correlation between in‐person gold‐standard full‐scale IQ and remotely administered Penn‐CNB Continuous Performance Task accuracy score. **(B)** Correlation between in‐person gold‐standard full‐scale IQ and remotely administered Penn‐CNB Matrix Reasoning accuracy score. **(C)** Correlation between in‐person gold‐standard full‐scale IQ and remotely administered Penn‐CNB global accuracy score, excluding the Continuous Performance Task.
**Figure S3:** Instructions for taking height and weight measurements.
**Figure S4:** Instructions for administering the VMI.
**Figure S5:** Decision tree illustrating Penn‐CNB quality control process. Adapted from Penn‐CNB manual.
**Table S2:** List of commonly prescribed psychiatric medications.

## Data Availability

The datasets used and/or analysed during the current study are available from the corresponding author on reasonable request.

## References

[jir70116-bib-0001] Achenbach, T. M. , and L. A. Rescorla . 2001. Manual for the ASEBA School‐Age Forms & Profiles. VT, University of Vermont, Research Center for Children, Youth and Families.

[jir70116-bib-0002] Achenbach, T. M. , and L. A. Rescorla . 2003. Manual for the ASEBA Adult Forms & Profiles. VT, University of Vermont, Research Center for Children, Youth and Families.

[jir70116-bib-0003] Addington, J. , L. Liu , L. Buchy , et al. 2015. “North American Prodrome Longitudinal Study (NAPLS 2): The Prodromal Symptoms.” Journal of Nervous and Mental Disease 203: 328–335. 25919383 10.1097/NMD.0000000000000290 PMC4417745

[jir70116-bib-0004] Ballif, B. C. , A. Theisen , J. Coppinger , et al. 2008. “Expanding the Clinical Phenotype of the 3q29 Microdeletion Syndrome and Characterization of the Reciprocal Microduplication.” Molecular Cytogenetics 1: 8. 18471269 10.1186/1755-8166-1-8 PMC2408925

[jir70116-bib-0005] Beery, K. E. , and N. A. Beery . 2010. The Beery‐Buktenica Developmental Test of Visual‐Motor Integration (Beery VMI): With Supplemental Developmental Tests of Visual Perception and Motor Coordination and Stepping Stones Age Norms From Birth to Age Six: Administration, Scoring, and Teaching Manual. Pearson.

[jir70116-bib-0006] Campbell, I. M. , S. E. Sheppard , T. B. Crowley , et al. 2018. “What Is New With 22q? An Update From the 22q and You Center at the Children's Hospital of Philadelphia.” American Journal of Medical Genetics. Part A 176: 2058–2069. 30380191 10.1002/ajmg.a.40637 PMC6501214

[jir70116-bib-0007] Coe, B. P. , H. A. F. Stessman , A. Sulovari , et al. 2019. “Neurodevelopmental Disease Genes Implicated by de Novo Mutation and Copy Number Variation Morbidity.” Nature Genetics 51: 106–116. 30559488 10.1038/s41588-018-0288-4 PMC6309590

[jir70116-bib-0008] Conners, C. K. 2022. Conners 4th Edition. Multi‐Health Systems.

[jir70116-bib-0009] Conners, C. K. , D. Erhardt , and E. Sparrow . 1999. Conners' Adult ADHD Rating Scales.

[jir70116-bib-0010] Conners, C. K. , S. R. Rzepa , J. Pitkanen , and S. Mears . 2018. “Conners 3rd Edition (Conners 3; Conners 2008).” In Encyclopedia of clinical neuropsychology. Springer.

[jir70116-bib-0011] Constantino, J. N. , and R. D. Todd . 2012. The Social Responsiveness Scale Manual. Western Psychological Services.

[jir70116-bib-0012] Cox, D. M. , and M. G. Butler . 2015. “A Clinical Case Report and Literature Review of the 3q29 Microdeletion Syndrome.” Clinical Dysmorphology 24: 89–94. 25714563 10.1097/MCD.0000000000000077 PMC5125389

[jir70116-bib-0013] Dunn, D. M. 2019. Peabody Picture Vocabulary Test. 5th ed. MN, NCS Pearson.

[jir70116-bib-0014] Elliott, C. D. , G. Murray , and L. Pearson . 1990. “Differential Ability Scales.” San Antonio, Texas.

[jir70116-bib-0015] Friedrich, S. , F. Konietschke , and M. Pauly . 2018. “Analysis of Multivariate Data and Repeated Measures Designs With the R Package MANOVA.” Results in Mathematics arXiv preprint arXiv:1801.08002.

[jir70116-bib-0016] Fu, Z. , Y. X. Jia , J. X. Fu , et al. 2021. “A Case of 15q11‐q13 Duplication Syndrome and Literature Review.” Brain and Behavior: A Cognitive Neuroscience Perspective 11: e2219. 10.1002/brb3.2219 PMC8413793 34292674

[jir70116-bib-0017] Gioia, G. , P. Isquith , S. Guy , and L. Kenworthy . 2015. Behavior Rating Inventory of Executive Function, Second Edition (BRIEF2). FL, PAR Inc.

[jir70116-bib-0018] Glassford, M. R. , J. A. Rosenfeld , A. A. Freedman , M. E. Zwick , J. G. Mulle , and Unique Rare Chromosome Disorder Support Group . 2016. “Novel Features of 3q29 Deletion Syndrome: Results from the 3q29 Registry.” American Journal of Medical Genetics. Part A 170A: 999–1006. 26738761 10.1002/ajmg.a.37537 PMC4849199

[jir70116-bib-0019] Grosse, S. D. , M. S. Schechter , R. Kulkarni , M. A. Lloyd‐Puryear , B. Strickland , and E. Trevathan . 2009. “Models of Comprehensive Multidisciplinary Care for Individuals in the United States With Genetic Disorders.” Pediatrics 123: 407–412. 19117908 10.1542/peds.2007-2875

[jir70116-bib-0020] Gur, R. C. , T. M. Moore , R. Weinberger , et al. 2021. “Relationship Between Intelligence Quotient Measures and Computerized Neurocognitive Performance in 22q11.2 Deletion Syndrome.” Brain and Behavior: A Cognitive Neuroscience Perspective 11: e2221. 10.1002/brb3.2221 PMC8413730 34213087

[jir70116-bib-0021] Harris, P. A. , R. Taylor , B. L. Minor , et al. 2019. “The REDCap Consortium: Building an International Community of Software Platform Partners.” Journal of Biomedical Informatics 95: 103208. 31078660 10.1016/j.jbi.2019.103208 PMC7254481

[jir70116-bib-0022] Harris, P. A. , R. Taylor , R. Thielke , J. Payne , N. Gonzalez , and J. G. Conde . 2009. “Research Electronic Data Capture (REDCap)‐A Metadata‐Driven Methodology and Workflow Process for Providing Translational Research Informatics Support.” Journal of Biomedical Informatics 42: 377–381. 18929686 10.1016/j.jbi.2008.08.010 PMC2700030

[jir70116-bib-0023] Kaminsky, E. B. , V. Kaul , J. Paschall , et al. 2011. “An Evidence‐Based Approach to Establish the Functional and Clinical Significance of Copy Number Variants in Intellectual and Developmental Disabilities.” Genetics in Medicine 13: 777–784. 21844811 10.1097/GIM.0b013e31822c79f9 PMC3661946

[jir70116-bib-0024] Katakis, P. , G. L. Estrin , J. Wolstencroft , et al. 2023. “Diagnostic Assessment of Autism in Children Using Telehealth in a Global Context: A Systematic Review.” Review Journal of Autism and Developmental Disorders 12: 516–566.

[jir70116-bib-0025] Kendall, K. M. , E. Rees , V. Escott‐Price , et al. 2017. “Cognitive Performance Among Carriers of Pathogenic Copy Number Variants: Analysis of 152,000 UK Biobank Subjects.” Biological Psychiatry 82: 103–110. 27773354 10.1016/j.biopsych.2016.08.014

[jir70116-bib-0026] Kirov, G. , A. J. Pocklington , P. Holmans , et al. 2012. “De Novo CNV Analysis Implicates Specific Abnormalities of Postsynaptic Signalling Complexes in the Pathogenesis of Schizophrenia.” Molecular Psychiatry 17: 142–153. 22083728 10.1038/mp.2011.154 PMC3603134

[jir70116-bib-0027] Klaiman, C. , S. P. White , C. Saulnier , et al. 2022. “A Distinct Cognitive Profile in Individuals With 3q29 Deletion Syndrome.” Journal of Intellectual Disability Research 67: 216–227. 35297118 10.1111/jir.12919

[jir70116-bib-0028] Levy, T. , B. Lerman , D. Halpern , et al. 2022. “CHAMP1 Disorder Is Associated With a Complex Neurobehavioral Phenotype Including Autism, ADHD, Repetitive Behaviors and Sensory Symptoms.” Human Molecular Genetics 31: 2582–2594. 35271727 10.1093/hmg/ddac018 PMC9396938

[jir70116-bib-0029] Loewy, R. L. , R. Pearson , S. Vinogradov , C. E. Bearden , and T. D. Cannon . 2011. “Psychosis Risk Screening With the Prodromal Questionnaire‐Brief Version (PQ‐B).” Schizophrenia Research 129: 42–46. 21511440 10.1016/j.schres.2011.03.029 PMC3113633

[jir70116-bib-0030] Malhotra, D. , and J. Sebat . 2012. “CNVs: Harbingers of a Rare Variant Revolution in Psychiatric Genetics.” Cell 148: 1223–1241. 22424231 10.1016/j.cell.2012.02.039 PMC3351385

[jir70116-bib-0031] Marshall, C. R. , D. P. Howrigan , D. Merico , et al. 2017. “Contribution of Copy Number Variants to Schizophrenia From a Genome‐Wide Study of 41,321 Subjects.” Nature Genetics 49: 27–35. 27869829 10.1038/ng.3725 PMC5737772

[jir70116-bib-0032] Miller, T. J. , T. H. Mcglashan , J. L. Rosen , et al. 2003. “Prodromal Assessment With the Structured Interview for Prodromal Syndromes and the Scale of Prodromal Symptoms: Predictive Validity, Interrater Reliability, and Training to Reliability.” Schizophrenia Bulletin 29: 703–715. 14989408 10.1093/oxfordjournals.schbul.a007040

[jir70116-bib-0033] Moore, T. M. , S. P. Reise , R. E. Gur , H. Hakonarson , and R. C. Gur . 2015. “Psychometric Properties of the Penn Computerized Neurocognitive Battery.” Neuropsychology 29: 235–246. 25180981 10.1037/neu0000093 PMC4345134

[jir70116-bib-0034] Mudigoudar, B. , S. Nune , S. Fulton , E. Dayyat , and J. W. Wheless . 2017. “Epilepsy in 22q11.2 Deletion Syndrome: A Case Series and Literature Review.” Pediatric Neurology 76: 86–90. 28969878 10.1016/j.pediatrneurol.2017.08.011

[jir70116-bib-0035] Mulle, J. 2015. “The 3q29 Deletion Confers > 40‐Fold Increase in Risk for Schizophrenia.” Molecular Psychiatry 20: 1028–1029. 26055425 10.1038/mp.2015.76 PMC4546529

[jir70116-bib-0036] Mulle, J. , A. Dodd , J. Mcgrath , et al. 2010. “Microdeletions of 3q29 Confer High Risk for Schizophrenia.” American Journal of Human Genetics 87: 229–236. 20691406 10.1016/j.ajhg.2010.07.013 PMC2917706

[jir70116-bib-0037] Mulle, J. G. , P. F. Sullivan , and J. Hjerling‐Leffler . 2021. “Editorial Overview: Rare CNV Disorders and Neuropsychiatric Phenotypes: Opportunities, Challenges, Solutions.” Current Opinion in Genetics & Development 68: iii–ix. 34059379 10.1016/j.gde.2021.05.002 PMC8722467

[jir70116-bib-0038] Murphy, M. M. , T. L. Burrell , J. F. Cubells , et al. 2020. “Comprehensive Phenotyping of Neuropsychiatric Traits in a Multiplex 3q29 Deletion Family: A Case Report.” BMC Psychiatry 20: 184. 32321479 10.1186/s12888-020-02598-w PMC7179007

[jir70116-bib-0039] Murphy, M. M. , T. Lindsey Burrell , J. F. Cubells , et al. 2018. “Study Protocol for the Emory 3q29 Project: Evaluation of Neurodevelopmental, Psychiatric, and Medical Symptoms in 3q29 Deletion Syndrome.” BMC Psychiatry 18: 183. 29884173 10.1186/s12888-018-1760-5 PMC5994080

[jir70116-bib-0040] Nissen, T. , and R. Wynn . 2014. “The Clinical Case Report: A Review of Its Merits and Limitations.” BMC Research Notes 7: 264. 24758689 10.1186/1756-0500-7-264 PMC4001358

[jir70116-bib-0041] O'hora, K. P. , Z. Zhang , A. Vajdi , et al. 2022. “Neurobehavioral Dimensions of Prader Willi Syndrome: Relationships Between Sleep and Psychosis‐Risk Symptoms.” Frontiers in Psychiatry 13: 868536. 35492689 10.3389/fpsyt.2022.868536 PMC9043455

[jir70116-bib-0042] Pinchefsky, E. , L. Laneuville , and M. Srour . 2017. “Distal 22q11.2 Microduplication: Case Report and Review of the Literature.” Child Neurology Open 4: 2329048x17737651. 10.1177/2329048X17737651 PMC5673001 29147671

[jir70116-bib-0043] Pollak, R. , T. Burrell , J. Cubells , et al. 2023. “Visual‐Motor Integration Deficits in 3q29 Deletion Syndrome.” Journal of Autism and Developmental Disorders 54: 3142–3154. 37354284 10.1007/s10803-023-06034-2 PMC11300491

[jir70116-bib-0044] R Core Team . 2008. R: A Language and Environment for Statistical Computing. R Foundation for Statistical Computing.

[jir70116-bib-0045] Ramoni, R. B. , J. J. Mulvihill , D. R. Adams , et al. 2017. “The Undiagnosed Diseases Network: Accelerating Discovery About Health and Disease.” American Journal of Human Genetics 100: 185–192. 28157539 10.1016/j.ajhg.2017.01.006 PMC5294757

[jir70116-bib-0046] Rees, E. , K. Kendall , A. F. Pardiñas , et al. 2016. “Analysis of Intellectual Disability Copy Number Variants for Association With Schizophrenia.” JAMA Psychiatry 73: 963–969. 27602560 10.1001/jamapsychiatry.2016.1831 PMC5014093

[jir70116-bib-0047] Roth, R. M. , and G. A. Gioia . 2005. Behavior Rating Inventory of Executive Function‐Adult Version. Psychological Assessment Resources.

[jir70116-bib-0048] Rutter, M. , A. Bailey , and C. Lord . 2003. The Social Communication Questionnaire ‐ Manual. Western Psychological Services.

[jir70116-bib-0049] Rutter, M. , A. Le Couteur , and C. Lord . 2003. Autism Diagnostic Interview‐Revised, 29–30. Western Psychological Services.

[jir70116-bib-0050] Sanchez Russo, R. , M. J. Gambello , M. M. Murphy , et al. 2021. “Deep Phenotyping in 3q29 Deletion Syndrome: Recommendations for Clinical Care.” Genetics in Medicine 23: 872–880. 33564151 10.1038/s41436-020-01053-1 PMC8105170

[jir70116-bib-0051] Sefik, E. , R. M. Guest , K. Aberizk , et al. 2024. “Psychosis Spectrum Symptoms Among Individuals With Schizophrenia‐Associated Copy Number Variants and Evidence of Cerebellar Correlates of Symptom Severity.” Psychiatry Research 335: 115867. 38537595 10.1016/j.psychres.2024.115867

[jir70116-bib-0052] Silver, H. , R. Greenberg , P. M. Siper , et al. 2025. “Protein‐Truncating Variants and Deletions of SHANK2 Are Associated With Autism Spectrum Disorder and Other Neurodevelopmental Concerns.” Journal of Neurodevelopmental Disorders 17: 25. 40307697 10.1186/s11689-025-09600-0 PMC12042525

[jir70116-bib-0053] Solomon, D. , and N. Soares . 2020. “Telehealth Approaches to Care Coordination in Autism Spectrum Disorder.” In Interprofessional care coordination for pediatric autism Spectrum disorder: Translating research into practice. Springer.

[jir70116-bib-0054] Sparrow, S. , D. Cicchetti , and C. Saulnier . 2016. Vineland Adaptive Behavior Scales–Third Edition (Vineland‐3). American Guidance Service.

[jir70116-bib-0055] Stefansson, H. , A. Meyer‐Lindenberg , S. Steinberg , et al. 2014. “CNVs Conferring Risk of Autism or Schizophrenia Affect Cognition in Controls.” Nature 505: 361–366. 24352232 10.1038/nature12818

[jir70116-bib-0056] Stroupková, L. , M. Vyhnalová , S. Kolář , et al. 2024. “Use of Telehealth in Autism Spectrum Disorder Assessment in Children: Evaluation of an Online Diagnostic Protocol Including the Brief Observation of Symptoms of Autism.” Journal of Autism and Developmental Disorders 56: 148–161. 39325287 10.1007/s10803-024-06524-x

[jir70116-bib-0057] Sullivan, P. F. , and M. J. Owen . 2020. “Increasing the Clinical Psychiatric Knowledge Base About Pathogenic Copy Number Variation.” American Journal of Psychiatry 177: 204–209. 32114777 10.1176/appi.ajp.2019.19040335 PMC7080301

[jir70116-bib-0058] Szatkiewicz, J. P. , C. O'dushlaine , G. Chen , et al. 2014. “Copy Number Variation in Schizophrenia in Sweden.” Molecular Psychiatry 19: 762–773. 24776740 10.1038/mp.2014.40 PMC4271733

[jir70116-bib-0059] Tang, S. X. , J. J. Yi , T. M. Moore , et al. 2014. “Subthreshold Psychotic Symptoms in 22q11.2 Deletion Syndrome.” Journal of the American Academy of Child and Adolescent Psychiatry 53: 991–1000.e2. 25151422 10.1016/j.jaac.2014.05.009 PMC4159384

[jir70116-bib-0060] Taylor, C. M. , B. M. Finucane , A. Moreno‐De‐Luca , L. K. Walsh , C. L. Martin , and D. H. Ledbetter . 2023. “Phenotypic Shift in Copy Number Variants: Evidence in 16p11.2 Duplication Syndrome.” Genetics in Medicine 25: 151–154. 36609147 10.1016/j.gim.2022.09.011 PMC10068678

[jir70116-bib-0061] Wechsler, D. 1999. “Wechsler Abbreviated Scale of Intelligence”.

[jir70116-bib-0062] Weinberger, R. , O. Weisman , Y. Guri , T. Harel , A. Weizman , and D. Gothelf . 2018. “The Interaction Between Neurocognitive Functioning, Subthreshold Psychotic Symptoms and Pharmacotherapy in 22q11. 2 Deletion Syndrome: A Longitudinal Comparative Study.” European Psychiatry 48: 20–26. 29331595 10.1016/j.eurpsy.2017.10.010

[jir70116-bib-0063] White, L. K. , N. Hillman , K. Ruparel , et al. 2024. “Remote Assessment of the Penn Computerised Neurocognitive Battery in Individuals With 22q11.2 Deletion Syndrome.” Journal of Intellectual Disability Research 68: 369–376. 38229473 10.1111/jir.13115 PMC13107926

[jir70116-bib-0064] Wickham, H. 2009. ggplot2: Elegant Graphics for Data Analysis. Springer‐Verlag.

[jir70116-bib-0065] Willatt, L. , J. Cox , J. Barber , et al. 2005. “3q29 Microdeletion Syndrome: Clinical and Molecular Characterization of a New Syndrome.” American Journal of Human Genetics 77: 154–160. 15918153 10.1086/431653 PMC1226188

[jir70116-bib-0066] Zaks, N. , B. Mahjani , A. Reichenberg , and R. Birnbaum . 2025. “Clinical and Cognitive Phenotyping of Copy Number Variants Associated With Neurodevelopmental Disorders From a Multi‐Ancestry Biobank medRxiv: The Preprint Server for Health Sciences.” 2024.07.16.24310489.

